# Collisional cross-section of water molecules in vapour studied by means of ^1^H relaxation in NMR

**DOI:** 10.1038/srep38492

**Published:** 2016-12-23

**Authors:** Daniele Mammoli, Estel Canet, Roberto Buratto, Pascal Miéville, Lothar Helm, Geoffrey Bodenhausen

**Affiliations:** 1 Institut des Sciences et Ingéniérie Chimiques, Ecole Polytechnique Fédérale de Lausanne, 1015 Lausanne, Switzerland; 2 Département de Chimie, Ecole Normale Superieure, PSL Research University, UPMC Univ Paris 06, CNRS, Laboratoire des Biomolecules (LBM), 24 rue Lhomond, 75005 Paris, France; 3 Sorbonne Universites, UPMC Univ Paris 06, Ecole Normale Superieure, CNRS, Laboratoire des Biomolecules (LBM), Paris, France

## Abstract

In gas phase, collisions that affect the rotational angular momentum lead to the return of the magnetization to its equilibrium (relaxation) in Nuclear Magnetic Resonance (NMR). To the best of our knowledge, the longitudinal relaxation rates *R*_1_ = 1/*T*_1_ of protons in H_2_O and HDO have never been measured in gas phase. We report *R*_1_ in gas phase in a field of 18.8 T, i.e., at a proton Larmor frequency *ν*_*0*_ = 800 MHz, at temperatures between 353 and 373 K and pressures between 9 and 101 kPa. By assuming that spin rotation is the dominant relaxation mechanism, we estimated the effective cross-section σ_*J*_ for the transfer of angular momentum due to H_2_O-H_2_O and HDO-D_2_O collisions. Our results allow one to test theoretical predictions of the intermolecular potential of water in gas phase.

Water is the most extensively studied molecule on Earth. A precise determination of its intermolecular potential would allow accurate predictions of its properties in gas, liquid and solid phase. However, despite huge theoretical efforts^1^,^2^, a full agreement with experiments^3^,^4^,^5^,^6^ has not yet been achieved. Nuclear magnetic resonance (NMR) of molecules in gas phase^7^ has some unique features. The coupling between nuclear spins and magnetic moments induced by molecular rotation implies that collisions between molecules lead to a relaxation, i.e. to the return of the longitudinal magnetization *M*_*z*_ to its equilibrium after a perturbation, through a mechanism known as spin-rotation. If spin-rotation is the dominant mechanism, cross-sections for the transfer of angular momentum can be obtained from NMR relaxation rates in gas phase. Such relaxation rates have been measured over a wide range of pressures and temperatures^8^,^9^,^10^. Experimentally determined cross-sections can be used to refine intermolecular potentials^11^,^12^,^13^. In methane, isotopic substitution^14^,^15^,^16^ affects relaxation rates associated with the different isotopomers such as CH_4_, CH_3_D, CH_2_D_2_, and CHD_3_. In supercritical water^17^,^18^,^19^, spin-rotation significantly contributes to NMR relaxation despite the high density. In the context of our attempts to prepare *para*-water^20^,^21^,^22^,^23^,^24^,^25^,^26^ and related spin states in other molecules^27^,^28^,^29^,^30^,^31^,^32^, we have measured longitudinal relaxation rates *R*_*1*_ = 1/*T*_*1*_ of gaseous H_2_O and HDO at different temperatures and pressures. To the best of our knowledge, this is the first time that such observations are reported. Our measurements are useful to refine intermolecular potentials for water vapour. These may be compared with water confined in matrices^33^,^34^ or in fullerene cages^21^,^35^ where a gas-phase like behaviour can be observed.

## Theory

Collisions between molecules can induce transitions between rotational quantum states. As a result, spin-dependent interactions vary as a function of time and, if the fluctuations occur at frequencies in the vicinity of the nuclear Larmor frequency ω_0_, longitudinal NMR relaxation takes place. Comprehensive theoretical treatments of NMR relaxation can be found elsewhere^36^,^37^,^38^,^39^,^40^,^41^,^42^,^43^,^44^. In this article, we shall only mention some aspects of spin-rotation and dipole-dipole relaxation mechanisms that are relevant to longitudinal relaxation in gas phase.

Spin-rotation (SR) relaxation is due to collisions that modulate local fields induced at the sites of the nuclei by the rotation of the surrounding electronic cloud.

Relaxation induced by spin-rotation can be described by ref. 45:


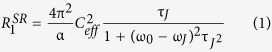


where:


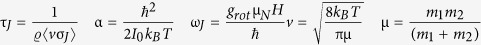


τ_*J*_ is the spin-rotation correlation time, *C*_*eff*_ (in Hz) the spin-rotation constant, ω_*J*_ the rotational frequency (in rad/s)^46^, 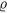 is the number density of molecules, *v* is the average thermal velocity, σ_*J*_ is the collisional cross-section for the transfer of angular momentum, *I*_0_ is the moment of inertia, *g*_*rot*_ is the g-factor, μ_*N*_ is the nuclear magneton, *H* is the magnetic field and μ is the reduced mass of the two colliding particles. The correlation time τ_*J*_ is related to the lifetime of the rotational quantum states. The relaxation process can be described by characterizing the cross-section for the transfer of angular momentum. Intermolecular potentials used to model the interaction mostly consist of an isotropic part, usually a radial function, depending only on the distance between particles (e.g., Lennard-Jones potential) and an anisotropic part, depending also on the orientation of the molecules with respect to each other. The intermolecular potential of a molecule can be written by considering its axial symmetry^47^ and can be linked to relaxation rates via the Bloom – Oppenheim theory^48^.

Dipole-dipole (DD) relaxation is due to fluctuations of the interaction between magnetic dipoles, which are induced by physical rotation. The DD interactions are described by a correlation time τ_C_ that is related to the mean time needed for the molecule to undergo a rotation through one radiant. DD relaxation can occur between spins in the same molecule (intramolecular DD) or between spins in different molecules (intermolecular DD). Relaxation by the intramolecular DD interaction between the two protons of water is described by ref. 36:


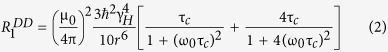


where *r* is the distance between the protons, *γ*_*H*_ is the gyromagnetic ratio of protons and μ_0_ is the magnetic permeability in vacuum.

In liquid phase, the rotational correlation time τ_*C*_ is linked to τ_*J*_ by the Hubbard relation^49^ τ_*C*_/τ_*J*_ = 1/6. For dilute gases (τ_*J*_ → ∞), the ratio of correlation times τ_*C*_/τ_*J*_ varies from 5/4 (Ivanov model) to 1/4 (extended diffusion model) or 1/24.4 (Langevin model)^14^,^15^.

## Results

We measured longitudinal relaxation rates *R*_*1*_ by the conventional inversion-recovery method. Experiments were carried out at temperatures *T* = 353, 363, and 373 K and pressures 9 < *p* < 101 kPa. The translational diffusion of water molecules does not affect our measurements of longitudinal relaxation rates *R*_*1*_ (see Methods), although it might interfere with measurements of transverse relaxation rates *R*_*2*_. The rates *R*_*1*_ observed in neat water (samples 1–4, H_2_O-H_2_O collisions) and in a mixture of HDO and D_2_O (sample 5, HDO-D_2_O collisions) are reported in Table 1.

**Figure 1:**
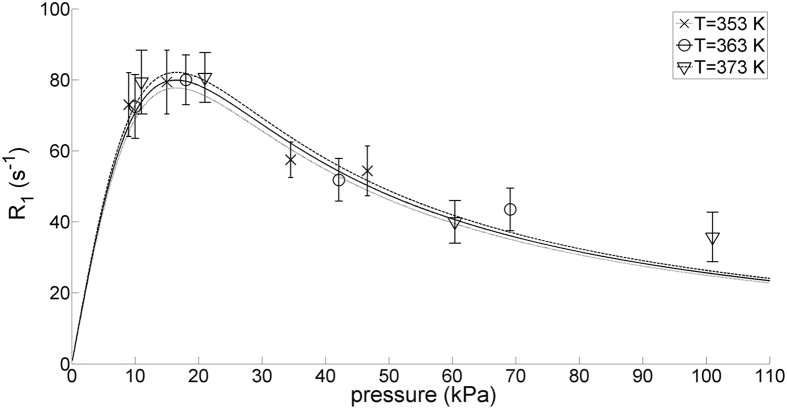
(Points) Experimental rates *R*_*1*_ of gaseous H_2_O at 800** **MHz and at pressures 9 < p < 101 kPa. (Lines) Estimates of *R*_*1*_ arising from spin-rotation, using Eq. 1 with the parameters in Table 2.

We shall initially consider spin-rotation to be the dominant relaxation mechanism, neglecting dipole-dipole relaxation. Under our experimental conditions, water vapour is mainly monomeric^50^,^51^,^52^ and the extreme narrowing regime 
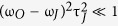
 is not fulfilled: *R*_*1*_ shows a maximum at a pressure *p*^max^ where τ_*J*_ = 1/(ω_*O*_ − ω_*J*_) (see Fig. 1).

The number density 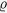 at pressure *p* can be estimated via the ideal gas law (see Methods) yielding τ_*J*_ = *RT*/(*pvσ*_*J*_). Hence, it is possible to calculate the cross-section 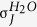 for H_2_O:H_2_O collisions at *p*^max^ as 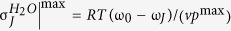. If we assume 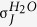 to be independent of *p* (i.e. 
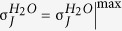
) at the low pressures used in our experiments, we can substitute 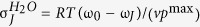 in 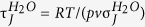 to find 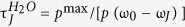. This last relationship can be used to predict the dependence of *R*_*1*_ on *p*, at a given *T*, by using Eq. 1 and the parameters in Table 2. All three curves result from fitting a single parameter (*p*^max^), all the other parameters being fixed to the values given in Table 2. The fitted value *p*^max^ = (17 ± 3) kPa provides a fair agreement between experimental relaxation rates (points) and predicted rates (lines) (Fig. 1). The spin-rotation tensor depends on the symmetry of the molecule: in our approximation we take into account only the isotropic constant *C*_*eff*_^35^,^53^,^54^ which we consider to be independent of both pressure and temperature. The fitted value of *p*^max^ is constant while *C*_*eff*_ is fixed to values comprised in its confidence range.

In a more refined analysis we included contributions 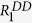 due to the intramolecular dipole-dipole interaction (Eq. 2). We fixed τ_*C*_ to values predicted by the Ivanov model (τ_*C*_ = 5/4τ_*J*_), extended diffusion model (τ_*C*_ = 1/4τ_*J*_) or Langevin model (τ_*C*_ = 1/24.4τ_*J*_)^14^,^15^. Our experimental data are compatible with a negligible dipole-dipole contribution or with the Langevin model (τ_*C*_ ≪ τ_*J*_), according to which significant contributions of 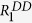 only occur at low pressures *p* < 10 kPa.

For HDO-D_2_O mixtures, experimental relaxation rates *R*_1_ (sample 5) are reported in Table 1. In this case, we can safely neglect DD contributions. The experimental rates *R*_*1*_ in Table 1 and the parameters in Table 2 are substituted into Eq. 1 to calculate the collision cross-sections.

Cross sections and correlation times for the transfer of the angular momentum in H_2_O:H_2_O and HDO:D_2_O collisions are reported in Table 3.

## Discussion

Our analysis provides information about H_2_O-H_2_O and HDO-D_2_O collisions at pressures below 101 kPa and temperatures between 353 and 373 K. The ratio of cross-sections 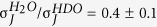 differs from the ratio of the moments of inertia 
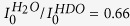
. This discrepancy suggests that there must be appreciable differences between the intermolecular potentials for HDO:D_2_O and H_2_O:H_2_O collisions. This hypothesis is compatible with the fact that H_2_O and D_2_O have almost equal electric dipole moments^55^ while the electric dipole moment of HDO differs in intensity and orientation from those of H_2_O and D_2_O^55^. NMR relaxation studies on the influence of hydrogen/deuterium isotopic substitution on collisional cross-sections have been reported for methane^15^,^16^. However, by isotopic substitution on methane only the moment of inertia is markedly altered. A direct comparison with isotopic substitution on the highly polar H_2_O is therefore not possible.

The collisional cross-sections calculated from our NMR data can be used to refine the anisotropic part of the intermolecular potentials for collisions in gas phase^56^,^57^,^58^ via the Bloom – Oppenheim theory^59^. However, such calculations are beyond the scope of this work.

Our findings may be relevant for Dissolution Dynamic Nuclear Polarization (D-DNP)^60^ where a frozen sample is rapidly heated by injecting a burst of superheated D_2_O (*T* > 373 K) into the cryostat, and the liquid HDO ‘bolus’, usually containing a hyperpolarized solute, is pushed by pressurized helium gas (typically at 1 MPa) through a polyethylene tube with a 1 mm inner diameter running through a “magnetic tunnel”^61^, with a length of ca. 4 m between the polarizer and the NMR or MRI system. Attempts to monitor the speed of the bolus moving through the tube by optical means have shown that it tends to break up into small droplets during the transfer. This increases the surface area where water molecules can exchange between the liquid and gaseous phases. If the liquid/gas exchange is fast, the *averaged* longitudinal relaxation rates are likely to be much shorter than those in liquid water. The shortening of *T*_*1*_ would lead to a rapid loss of hyperpolarization during the transfer between the polarizer and the NMR magnet. Note that the viscosity and surface tension of the transferred liquid are difficult to control, since it consists of an aqueous solution containing analytes, polarizing agents like TEMPOL and glass-forming agents such as glycerol.

To summarize, we reported NMR relaxation rates due to binary H_2_O:H_2_O and HDO:D_2_O collisions in the gas phase and evaluated the cross-sections for the transfer of the angular momentum which can be used to refine the intermolecular potentials.

## Methods

**Figure 2:**
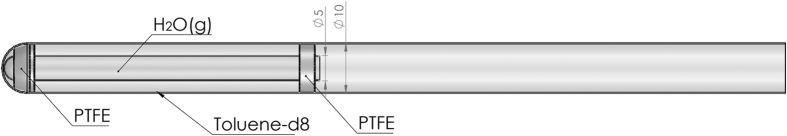
Schematic view of the coaxial tubes: outer tube (10 mm outer diameter) filled with toluene-d8 and inner tube (5 mm outer diameter, held by Teflon spacers) containing water vapour sealed under vacuum.

Our experimental setup consisted of a pair of coaxial glass tubes (Fig. 2).

**Figure 3:**
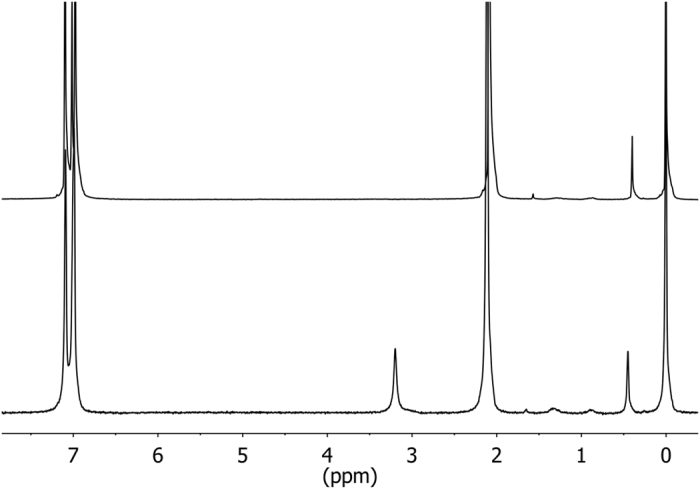
Proton NMR spectra of samples described in Fig. 2 at 800** **MHz and at 300** **K (top) or 363** **K (bottom). The signals at 2.1 and 7 ppm are attributed to residual protons in incompletely deuterated toluene-d8. The signal at 3.2 stems from water in gas phase. Small peaks between 0.3 and 2 ppm are due to impurities in toluene-d8. We diluted TMS in toluene-d8 to use its resonance at 0 ppm as chemical shift reference.

The inner tube with 5 mm outer diameter was held in the center of a 10 mm tube by holders made of PTFE (Teflon). The outer tube contained about 2 mL of deuterated toluene-d8 (boiling point *T*_*bp*_ = 384 K). Its deuterium signal allows one to lock the static field and to shim its homogeneity. The inner tube contained water that was frozen and flame-sealed under vacuum (*p* = 1 kPa). Four tubes of 3.5 to 4 cm length, labeled as samples 1, 2, 3 and 4, were filled with *ca* 0.1, 0.2, 0.3 and 4.5 mg H_2_O, determined with a precision balance (±0.1 mg, max. tara 31 g). A fifth tube (sample 5) was filled with 2 mg of 98% D_2_O and 2% H_2_O (*v*:*v*), hence containing *ca*. 2% HDO. The inner tube was completely immersed in the solvent contained in the outer tube (Fig. 2) in order to have a homogeneous temperature and to avoid condensation of water on the walls of the inner tube in regions outside the area where the temperature is accurately controlled. Before and after inserting the samples into the spectrometer, the temperature in the probe was determined with a platinum PT-100 resistance thermometer (“iTRON 08” by JUMO)^62^ using a similar set of two concentric tubes with toluene-d8 in the outer tube. After each experiment the maximum temperature variations were ±1 K. Two typical ^1^H NMR spectra are shown in Fig. 3: the peak near 3.2 ppm (w.r.t. TMS) is due to water in the gas phase at *T* = 363 K.

### NMR instrumentation

All NMR experiments have been performed on a Bruker Avance-II 800 MHz spectrometer equipped with a 10 mm BBO probe.

### Evaluation of pressure and density

To determine the pressure *p* and the number density 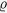 of the water in samples 1 to 5 we measured the mass of water and estimated the volume of the inner tubes. Samples 4 and 5 contain saturated vapour (*p* = *p*^sat^). In that case the pressure *p*^sat^ can be calculated using Antoine’s equation^63^:


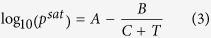


where *T* is the temperature and A, B and C are sample-specific constants. When expressing the pressure in bar and the temperature in K, we assumed^64^ A = 5.08354, B = 1663.125 and C = −45.622 for both H_2_O and D_2_O, since their vapour pressures are similar within 1% over our range of temperatures^65^. The number density 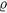 at a pressure *p* can be estimated provided that the equation of state of the gas is known *a priori*. We have compared 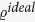 predicted by the ideal gas law with 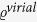 obtained from a second-order virial expansion. The deviation 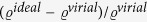 is always below 2% in the range of pressures and temperatures under investigation, so that the use of the ideal gas law is legitimate.

The quantity of water vapour in samples 1 to 3 has been determined by integration of the relevant signals in the NMR spectra. As a reference for integration we added 1,1,2,2-tetrachloroethane (C_2_H_2_Cl_4_, 0.2% *v*:*v*) to the toluene-d8 in the outer sample tube. We calibrated the integral of the C_2_H_2_Cl_4_ reference peak (near ~ 6 ppm) with respect to the number density of sample 4 (saturated vapour). The pressures in samples 1 to 3 are then determined by scaling the peak intensities of the vapour peak with respect to sample 4. The error on the pressures is assumed to be ±10%. The active volume of the 5 mm inner tube has been estimated from documentation by the manufacturer (Wilmad) to be 0.4 cm^3^.

### Translational diffusion and convection

Translational diffusion of water molecules in gas phase is very fast. Translational motion of water molecules between the active volume of the ^1^H NMR coil and the space outside the coil can affect inversion-recovery measurements of *T*_*1*_ relaxation. Indeed, molecules that carry inverted magnetization −*M*_*z*_ = −*M*_*z*_^*eq*^ within the active volume may be contaminated with molecules than come from areas outside the rf coil that carry magnetization in equilibrium M_z_^eq^ that has not been inverted. To ascertain the relevance of these effects on the time scale of the *T*_*1*_ measurement (max. 5 · *T*_*1*_ = 140 ms) we performed the following test. The inner tubes were only a few mm longer than the active region of the ^1^H coil of the 10 mm probe which is about 3 cm long. We measured *R*_1_ at the highest temperature *T* = 373 K (where the effects of diffusion are most pronounced) in two arrangements. First, we centered the inner tube with respect to the active region of the ^1^H coil. In this configuration, molecules can diffuse to and from the volumes above and below the active region. Secondly, we moved the inner tube up so that its bottom end was aligned with the lower end of the active region of the coil. In this manner, only molecules that cross the limit of the active region of the *rf* coil from above can influence the NMR signal. Any difference in *R*_*1*_ observed with these two configurations should be due to diffusion or convection. We found the *R*_*1*_ values to be identical within their errors, suggesting that contributions from diffusion can be neglected. Since we immersed the inner tube completely in a liquid with a controlled temperature, we assumed that there was no significant temperature gradient, so that convection due to differences in density should be negligible. Nevertheless, the experimental errors of the relaxation rates were doubled to take into account uncertainties stemming from diffusion and convection.

## Additional Information

**How to cite this article**: Mammoli, D. *et al*. Collisional cross-section of water molecules in vapour studied by means of ^1^H relaxation in NMR. *Sci. Rep.* **6**, 38492; doi: 10.1038/srep38492 (2016).

**Publisher's note:** Springer Nature remains neutral with regard to jurisdictional claims in published maps and institutional affiliations.

## Figures and Tables

**Table 1:** Longitudinal relaxation rates *R*_1_ for gaseous H_2_O (samples 1 to 4) and gaseous HDO (sample 5) at 800 MHz and at different temperatures and pressures.

Samples	H_2_O in H_2_O								HDO in D_2_O	
	1		2		3		4		5	
*T* (K)	*p* (kPa)	*R*_*1*_ (s^−1^)	*p* (kPa)	*R*_*1*_ (s^−1^)	*p* (kPa)	*R*_*1*_ (s^−1^)	*p* (kPa)	*R*_*1*_ (s^−1^)	*p* (kPa)	*R*_*1*_ (s^−1^)
353	9	73 ± 9	15	79 ± 9	34	57 ± 5	47	54 ± 7	47	57 ± 8
363	10	72 ± 9	18	80 ± 7	42	52 ± 6	69	43 ± 6	69	42 ± 5
373	11	79 ± 9	21	80 ± 7	60	40 ± 6	101	36 ± 7	101	31 ± 3

**Table 2:** Parameters used to calculate cross-sections via Eq. 1.

	*C*_*eff*_ (kHz)	ω_*0*_(rad/s)	*ω*_*J*_ (rad/s)	μ (kg)	*I*_*0*_ (kg·m^2^)
HDO	42.8 ± 0.1(a)	5 · 10^9^	6 · 10^8^(b)	1.64 · 10^−26^	2.91 · 10^−47^
H_2_O	32.2 ± 0.5(a)	5 · 10^9^	6 · 10^8^(b)	1.51 · 10^−26^	1.94 · 10^−47^

^(a)^Ref. 53

^(b)^Ref. 66.

**Table 3:** Correlation times τ_*J*_ and cross-sections σ_*J*_ for the angular momentum transfer in H_2_O:H_2_O and HDO:D_2_O collisions, calculated with Eq. 1 and parameters in Table 2.

*T* (K)	Cross-section σ_*J*_ (Å^2^)		Correlation time τ_*J*_(ps)	
	H_2_O:H_2_O collisions	HDO:D_2_O collisions	H_2_O:H_2_O collisions	HDO:D_2_O collisions
353 K	140 ± 26	378 ± 49	82 ± 15	32 ± 5
363 K	142 ± 26	367 ± 42	56 ± 10	22 ± 3
373 K	144 ± 27	354 ± 31	38 ± 7	16 ± 2
